# Cyclic Peptide MV6, an Aminoglycoside Efficacy Enhancer Against *Acinetobacter baumannii*

**DOI:** 10.3390/antibiotics13121147

**Published:** 2024-12-01

**Authors:** Natalia Roson-Calero, Jimmy Lucas, María A. Gomis-Font, Roger de Pedro-Jové, Antonio Oliver, Clara Ballesté-Delpierre, Jordi Vila

**Affiliations:** 1 Barcelona Institute for Global Health (ISGlobal), 08036 Barcelona, Spain; natalia.rc96.nr@gmail.com (N.R.-C.); jimmy.lucas@isglobal.org (J.L.); roger.depedro@isglobal.org (R.d.P.-J.); 2 Department of Basic Clinical Practice, School of Medicine, University of Barcelona, 08036 Barcelona, Spain; 3 CIBER de Enfermedades Infecciosas (CIBERINFEC), Instituto Salud Carlos III, 28029 Madrid, Spain; mariaantonia.gomis@ssib.es (M.A.G.-F.); antonio.oliver@ssib.es (A.O.); ccballes@clinic.cat (C.B.-D.); 4 Department of Microbiology, Hospital Universitario Son Espases, Health Research Institute of the Balearic Islands (IdISBa), 07120 Palma de Mallorca, Spain; 5 Department of Clinical Microbiology, Biomedical Diagnostic Center, Hospital Clinic, 08036 Barcelona, Spain

**Keywords:** antimicrobial peptides, antimicrobial resistance, *Acinetobacter baumannii*, adjuvants, efflux pump inhibition, aminoglycosides, MV6

## Abstract

**Background/Objectives**: *Acinetobacter baumannii* is a globally emerging pathogen with widespread antimicrobial resistance driven by multiple mechanisms, such as altered expression of efflux pumps like AdeABC, placing it as a priority for research. Driven by the lack of new treatments, alternative approaches are being explored to combat its infections, among which efficacy-enhancing adjuvants can be found. This study presents and characterizes MV6, a synthetic cyclic peptide that boosts aminoglycoside efficacy. **Methods**: MV6’s activity was assessed through antimicrobial susceptibility testing in combination with different antibiotic classes against *A. baumannii* strains characterized by PCR and RT-qPCR. PAβN served as a reference efflux pump inhibitor. Synergy was evaluated using checkerboard assays, and spontaneous mutants were generated with netilmicin with/without MV6 (100 mg/L). Whole-genome sequencing and variant calling analysis were then performed. **Results**: MV6 presented low antimicrobial activity in *A. baumannii* with MICs higher than 2048 mg/L. MV6 showed a better boosting effect for aminoglycosides, especially netilmicin, exceeding that of PAβN. Checkerboard assays confirmed a strong synergy between netilmicin and MV6, and a significant correlation was found between netilmicin MIC and *adeB* overexpression, which was mitigated by the presence of MV6. MV6 reduced, by 16-fold, the mutant prevention concentration of netilmicin. Mutations in a TetR-family regulator and ABC-binding proteins were found in both groups, suggesting a direct or indirect implication of these proteins in the resistance acquisition process. **Conclusions**: MV6 lacks intrinsic antimicrobial activity, minimizing selective pressure, yet enhances netilmicin’s effectiveness except for strain 210, which lacks the AdeABC efflux pump. Resistant mutants indicate specific aminoglycoside resistance mechanisms involving efflux pump mutations, suggesting synergistic interactions. Further research, including transcriptomic analysis, is essential to elucidate MV6’s role in enhancing netilmicin efficacy and its resistance mechanisms.

## 1. Introduction

*Acinetobacter baumannii* is a globally emerging opportunistic pathogen, notable for its broad range of antimicrobial resistance (AMR) mechanisms, which confer resistance to all classes of antimicrobials including last-resort carbapenems, therefore becoming a pan-drug resistant bacteria [1,2]. Its adaptability is driven by two key factors: (i) the acquisition of foreign resistance-conferring elements, such as transposons, plasmids, and resistance islands; and (ii) the regulation of innate resistance mechanisms, allowing for it to survive under selective pressure in the environment [1,3,4]. The threat of AMR in *A. baumannii* has positioned it as a critical priority for research investment and control efforts [5]. It is now recognized as a significant nosocomial pathogen, ranking among the top five pathogens responsible for AMR-related deaths globally, which may be recovered from bloodstream infections, ventilator-associated pneumonia, wound infections, urinary tract infections (UTIs), or meningitis [6,7,8,9].

*A. baumannii* can acquire AMR through horizontal gene transfer (HGT) via plasmids, insertion sequences (IS), and other mobile genetic elements. Additionally, it can develop resistance through spontaneous mutations that affect endogenous genes associated with membrane permeability or the expression of key resistance and transport proteins [10]. Efflux pumps play a crucial role in the clinically relevant pathogenicity and resistance profiles of this bacterium, as they actively expel multiple substrates from the cell, including a diverse array of antimicrobial agents [11]. The resistance-nodulation-cell division (RND) superfamily of transporters is notable in *A. baumannii*, with AdeABC and intrinsic AdeIJK as its main representatives [12,13]. Overproduction of these two is reportedly associated with an increase in resistance that acts synergistically with additional mechanisms, particularly the ones improving the permeability barrier for efflux pumps’ specific substrates. Therefore, there is an interplay between lower permeability associated with porin(s) deficiency and overproduction of efflux pumps [14]. Other efflux pumps belonging to different families, such as CraA, AbeM, and TetA/B, also contribute to multidrug resistance (MDR) [15,16,17]. Considering the high prevalence of genes encoding resistance enzymes, particularly aminoglycoside-modifying enzymes (AMEs), extended-spectrum β-lactamases (ESBLs), and carbapenemases in *A. baumannii*, it is becoming a particularly challenging pathogen to treat [1,18].

The current pipeline for anti-*Acinetobacter* treatments reveals a concerning lack of new-in-class antimicrobial agents nearing market approval [19,20]. During the decade from 2010 to 2019, only twelve antimicrobials were approved for market entry [21], of which only a few are viable options for treating *A. baumannii* infections. The approved agents include Cefiderocol [22], Eravacycline [23,24], Plazomicin [25], and Sulbactam/Durlobactam [26]. Recent attention has also been directed toward novel, yet non-approved, compounds targeting *A. baumannii*, including carbapenem-resistant strains. Some examples are GT-1, a siderophore-cephalosporin [27]; DS-8587, a broad-spectrum quinolone [28]; and AIC499, a β-lactam combined with a β-lactamase inhibitor [29]. Among the most promising novel antimicrobials is Abaucin, an antibiotic recently discovered through artificial intelligence that disrupts lipoprotein trafficking and exhibits a narrow spectrum of activity limited to *A. baumannii* [30].

In light of the notable lack of new treatments against this species, alternatives have arisen. The use of bacteriophages has shown success in mice [31,32], and several successful case studies involving phages have been reported [29]. Monoclonal antibodies are also studied for pneumonia and sepsis prevention [33,34]. Furthermore, the development of agents that reverse resistance mechanisms and restore susceptibility is actively being investigated. A typical example of this approach is the inhibition of β-lactamases; however, other strategies, such as efflux pump inhibitors (EPIs), also represent promising avenues for research [35]. Different EPIs, such as phenylalanine-arginine β-naphthylamide (PAβN), carbonyl cyanide-m-chlorophenylhydrazone (CCCP), and reserpine, have been identified. However, their use is currently limited to research purposes, as they show toxicity at therapeutic levels [36].

In this study, we present a synthetic cyclic peptide named MV6, which is able to resensitize bacteria to specific antibiotics. We investigate MV6’s potential application by examining its mechanisms of action and the genetic alterations present in spontaneous resistant mutants.

## 2. Results

### 2.1. MV6 Structure

**Figure 1:**
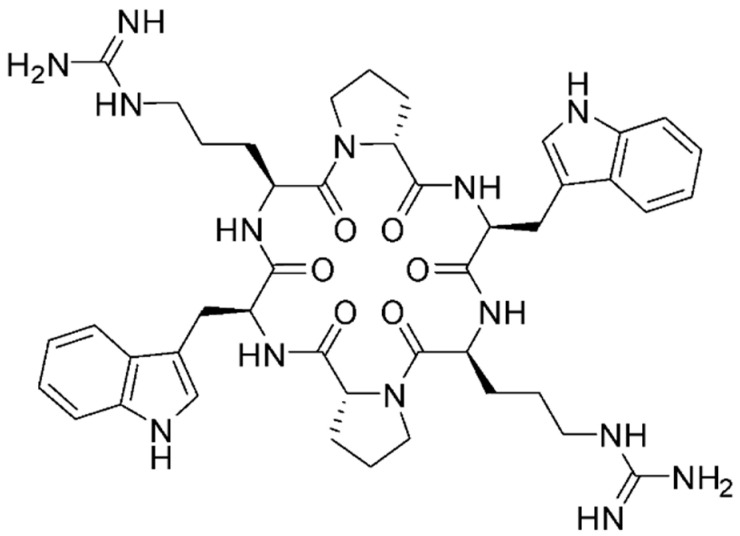
Chemical structure of MV6 cyclic peptide.

The MV6 cyclic peptide was selected for further study against *A. baumannii* from a synthetic library of 28 cyclic peptides following a small-scale “shot in the dark” approach in which each peptide was tested in combination with various antibiotics and bacterial species. Its structure consists of six amino acids, two arginine residues (Arg), two D-proline residues (D-Pro), and two tryptophan residues (Trp), arranged in a cyclic configuration. The final structure is &Arg-D-Pro-Trp-Arg-D-Pro-Trp& (Figure 1).

### 2.2. Strains’ Resistance Mechanisms

A high prevalence of AME among the selected *A. baumannii* strains was observed. The *aacC1* gene was the most prevalent, found in strains 80, 81, CR17, and CS01, and in some cases, it was the only AME detected. Strain 210 contained the highest number of AME-coding genes, including *aacC2*, *aphA6*, and *aphA1*. The *aadA1* gene was present in strains 80 and 81. Surprisingly, strain 306 did not exhibit any AME-related genes. Regarding efflux pump-related genes, all strains presented *tetB*, but none had *tetA*. The genes *adeJ* and *adeG* were present in all strains and so was *adeB* except for strain 210, which tested negative. The complete PCR results together with the corresponding protein products and expected substrates are listed in Table 1.

**Figure 2:**
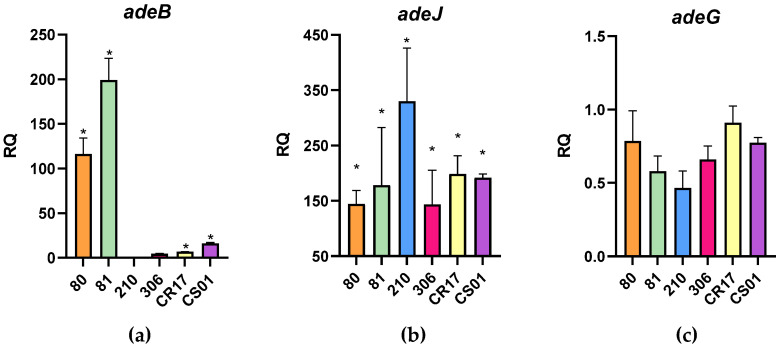
Expression levels of *adeB* (**a**), *adeJ* (**b**), and *adeG* (**c**) efflux pump genes (RQ) in selected *A. baumannii* strains. The symbol (*) indicates statistically significant overexpression of the corresponding gene in the specified strain (*p*-value < 0.05). All *p*-values showing statistical significance for *adeB* overexpression were <0.0001. *A. baumannii* ATCC 17978 was used as the reference strain, with *rpoB* and *gyrB* genes serving as internal controls for basal expression.

RT-qPCR revealed a statistically significant overexpression of *adeJ* in all strains (*p*-value < 0.05), with the highest relative quantification (RQ) values compared to the other genes tested. Although *adeG* was present in all strains, only constitutive expression was detected, with RQ values lower than 1. The expression of *adeB* varied across strains, with RQ values ranging from 15 to 200. Despite these differences, all strains except for strain 306 exhibited statistically significant overexpression of *adeB*. RT-qPCR also confirmed the absence of *adeB* in *A. baumannii* strain 210. All RQ values for *adeB*, *adeJ*, and *adeG* are shown in Figure 2.

### 2.3. Antimicrobial Susceptibility Testing and Checkerboard Assays

**Figure 3:**
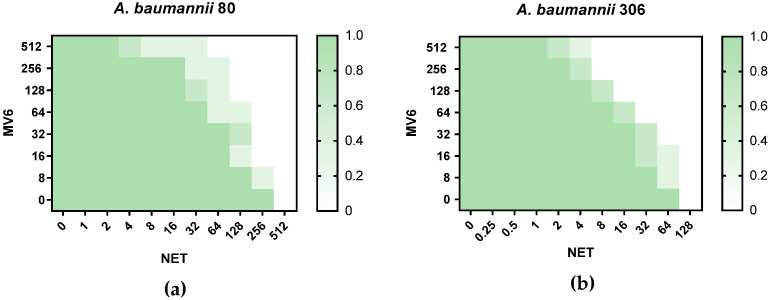
MIC distribution (mg/L) of NET and MV6 on the checkerboard assays. The plate figure ranges from 0, where no growth is observed, to 1.0, where growth was detected in all biological replicates. (**a**) Checkerboard assays for strain 80. (**b**) Checkerboard assays for strain 306.

Neither MV6 nor the efflux pump inhibitor PAβN exhibited growth inhibition at the concentrations used in this study. MICs for MV6 and PAβN were in all cases >2048 mg/L and 512 mg/L, respectively. All strains were able to tolerate 12.5% DMSO, making it feasible to use as a solvent, as the maximum concentration reached in the MIC plate was 0.5%. The results from combined susceptibility testing revealed that the MV6 peptide enhances the activity of aminoglycosides, particularly netilmicin (NET). No activity-boosting effect of MV6 was observed with other classes of antimicrobials. MV6 (100 mg/L) reduced the MIC of NET by 8- to 4-fold except for strain 210, in which the reduction was not significant. When compared to PAβN, the resensitizing activity of MV6 was slightly superior: strains 80 and 81 had a NET MIC of 256 mg/L, which was reduced to 128–64 mg/L with PAβN and to 32 mg/L in the presence of MV6 (Table 2). When treated with MV6, four out of six strains showed a reduction in NET’s MIC below the resistance breakpoint established by CLSI, lowering it to the intermediate category. The checkerboard assays revealed a FICI value of 0.0097 for strain 80 and of 0.0166 for strain 306, in both cases indicating a synergistic effect of MV6 over NET (Figure 3).

**Figure 4:**
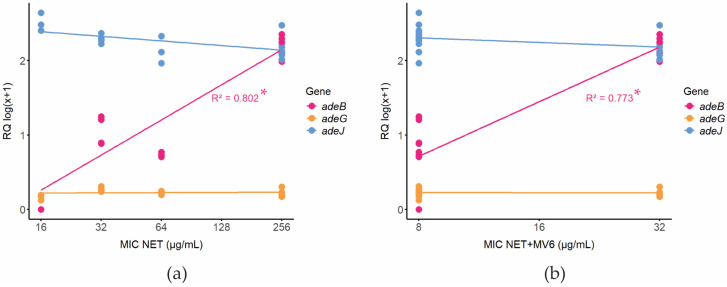
Linear regression analysis of *adeB* expression versus the MIC of NET alone and combined with MV6 peptide. (**a**) Correlation of *adeB*, *adeG*, and *adeJ* expression and NET’s MIC (* *p*-value = 5.089 × 10^−7^) (**b**) Correlation of *adeB*, *adeG*, and *adeJ* expression and NET’s MIC in presence of MV6 100 mg/L (* *p*-value = 1.552 × 10^−6^).

The analysis of the correlation between the increasing NET MIC values among the studied *A. baumannii* strains and the expression levels of the *adeB* gene indicated that, for both the NET and NET/MV6 datasets, *adeB* expression is significantly related to the log2(MIC) values, as shown in Figure 4. Moreover, the R^2^ values, 0.802 and 0.773 for NET and NET/MV6, respectively, suggest a strong relationship between the two parameters. The estimated intercept coefficients of 0.4678436 and 0.225576, respectively, represent the expected *adeB* expression values for a MIC = 0, with an increase in the logarithmic scale of *adeB* expression by 0.0067306 and 0.061134, respectively, for each treatment.

### 2.4. Resistance Profile Characterization of Spontaneous Mutants

**Figure 5:**
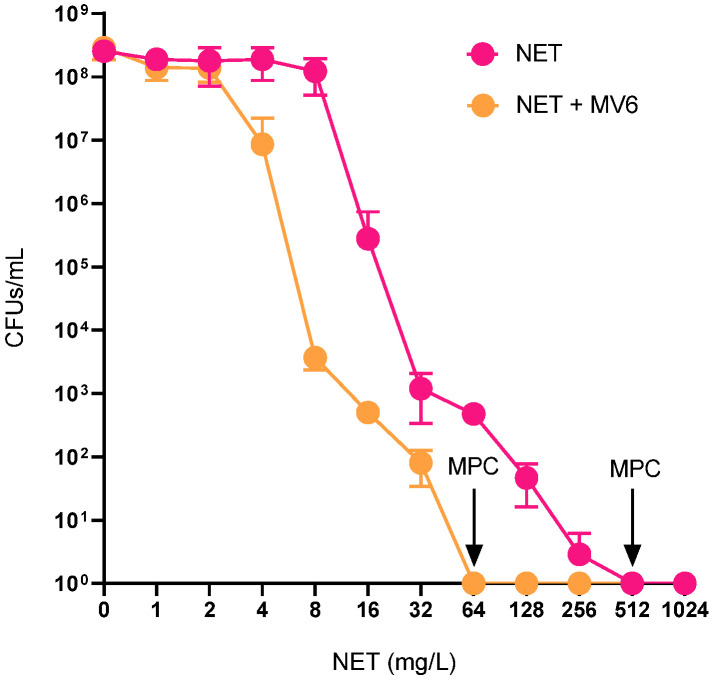
CFU/mL recount of mutant spontaneous generation for NET treatment and NET (+MV6 100 mg/L) combination treatment. The first concentration with no CFU recovered determines the mutant prevention concentration (MPC).

Population studies, as illustrated in Figure 5, demonstrate that the mutant prevention concentration (MPC) is significantly reduced when NET is combined with 100 mg/L of MV6. This combination inhibited the emergence of resistant mutants at 64 mg/L, whereas NET alone limited resistant mutant generation at 512 mg/L. Therefore, the presence of MV6 reduces the MPC by 8-fold. For perspective, the MIC for the combination and for NET alone was determined at 16 mg/L and 64 mg/L, respectively. Analysis of the resistance mechanisms in ten spontaneous mutants obtained from various resistance levels revealed, as expected, resistance to NET (Table 3). In the NET/MV6 mutant group, the median MIC to NET stands between 512 and 1024 mg/L, which decreased to 256 mg/L when MV6 was added. In contrast, in the NET-generated mutants, the median MIC for NET was 2048 mg/L, again dropping to 256 mg/L in the presence of MV6. The addition of MV6 resulted in an average 8-fold reduction in MIC for NET/MV6 compared to NET alone. No significant differences in the degree of MIC reduction were observed between the two groups.

**Figure 6:**
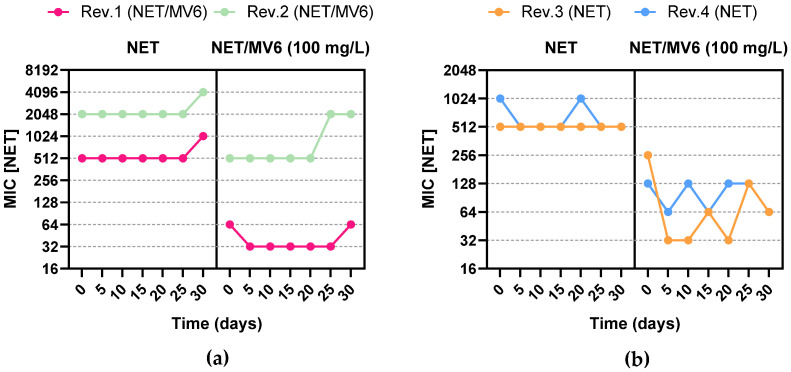
The 30-day analysis of NET MIC variation with and without MV6 for 4 different mutants. MICs for NET and NET/MV6 are represented in a time-lapse of 5 days. (**a**) Rev.1 and Rev.2 are spontaneous mutants generated with NET + MV6 100 mg/L; (**b**) Rev.3 and Rev.4 are spontaneous mutants generated with NET.

The results of microdilution assays using DKNGM Sensititre plates revealed the resistance profiles of the selected mutants. As expected, NET was not the only aminoglycoside impacted; variations in the MICs for tobramycin, amikacin, and gentamicin were also observed. This confirms that the mutation conferring resistance to NET affected resistance mechanisms involved in aminoglycoside resistance more broadly. No other significant cross resistances were observed for antibiotics belonging to other classes. The 30-day mutant reversion study demonstrated that the mutations conferring resistance to NET, whether generated in combination with MV6 or through monotherapy, were largely stable. Only one strain, generated with NET alone (Rev.3), exhibited a reduction in the MIC for NET/MV6, decreasing from 256 mg/L to 64 mg/L, representing a 4-fold reduction in MIC. However, no significant changes in the MIC for NET were observed in any of the four mutants tested over the 30-day experiment. Consequently, the original resistance levels were not regained, showing stable mutations across time, as represented in Figure 6.

### 2.5. Prediction of Resistance Mechanisms

Whole-genome sequencing (WGS) revealed recurrent mutations across the spontaneous mutants. As expected, mutations were identified in genes associated with resistance to aminoglycosides and regulators, especially those regulating the expression of efflux pumps. In the group of NET-generated mutants, alterations were identified in genes encoding ATP-binding proteins, TetR family regulators, and TetR-AcrR-like regulators as well as in the intergenic region between *adeR* (part of the AdeRS two-component system) and *adeA* (which encodes the membrane fusion protein of the efflux pump) within the *ade* operon responsible for expressing the AdeABC efflux pump. Additionally, mutations in various hypothetical proteins were detected, suggesting a potential, yet unknown, role of these genes in aminoglycoside resistance. Contrary to expectations, mutants generated from exposure to NET/MV6 exhibited similar mutations, including those affecting regulators of efflux pump systems. However, one exception was observed: a mutated tyrosyl-tRNA synthetase found in strain AB21, which was generated in NET at 128 mg/L. This mutation was not detected in any members of the NET/MV6 group. No other known resistance mechanisms were identified.

Most of the mutations exhibited a moderate to high impact, suggesting a potentially significant contribution to the resistance profile of the mutants. The most prevalent type of mutations were single nucleotide polymorphisms (SNPs), although insertions and deletions (INDELs) were also detected. Based on the results, high-impact mutations are typically associated with frameshift variants, moderate-impact mutations are commonly linked to missense variants, and low-impact mutations are mostly found in intergenic regions. All high-impact mutations correspond to a mutated TetR family gene (GBFHJJIP_01215). Table 4 lists the identified mutations for each mutant, their respective nature, and reference.

## 3. Discussion

Antimicrobial peptides (AMPs) represent a promising alternative for the treatment of *A. baumannii* in the emerging post-antibiotic era. AMPs typically attack bacterial membranes, which form the basis of their anti-*A. baumannii* activity, although some AMPs have been shown to act intracellularly as well. A well-known example is the human cathelicidin LL-37, a 37-amino acid AMP, whose primary antimicrobial mechanism involves the neutralization of lipopolysaccharides (LPS) in the bacterial outer membrane [44,45]. Nonetheless, an alternative use for AMPs has been explored, involving the enhancement of antibiotic efficacy by combining them with short peptides, which may or may not be conjugated to the antibiotic [46,47]. The six-amino acid peptide MV6 follows this approach by acting as an adjuvant that enhances the activity of other antimicrobials, specifically aminoglycosides. Lacking intrinsic antimicrobial activity, shown by MICs over 2048 mg/L, MV6 reduces selective pressure from antibiotics and minimizes the rate of spontaneous mutations, as demonstrated by MPC determinations. However, the generated mutations appear stable over long periods, with no reversion observed, which may pose a limitation to address in future development stages.

Among all the antimicrobials tested in combination with MV6, only aminoglycosides showed a decrease in MICs, particularly netilmicin, which revealed the higher fold changes in MIC reductions. This suggested a potential interaction between MV6 and the mechanism responsible for aminoglycoside resistance, which warranted further exploration. Regression analyses indicate a correlation between the expression levels of *adeB* and the MIC values of netilmicin, with MICs increasing in proportion to *adeB* overexpression. Although the addition of MV6 did not disrupt this correlation, it effectively reduced the MICs in strains with elevated *adeB* expression. A larger sample size will be required to explore this relationship in greater depth. MV6 at 100 mg/L manages to limit the increase in MIC to 32 mg/L, a concentration that could be reduced with a higher MV6 concentration, as demonstrated by the checkerboard assays. To highlight, strain 210 was the only one showing no significant effect of the MIC when combined with MV6 and was the only one lacking the AdeABC efflux pump. This finding reinforces the premise of a potential relationship between MV6 activity and *adeABC* expression, which will be further investigated. The strong synergistic effect of MV6 on netilmicin, as shown by FICI values considerably below the synergy threshold of 0.5, along with its low antimicrobial activity provide room to increase the MV6 dose and achieve a greater effect in enhancing netilmicin’s activity, achieving concentrations below the susceptibility breakpoint. Future toxicity, cytotoxicity, and hemolysis assays will be essential to define the therapeutic window for safe and effective use of MV6. Additionally, in vivo efficacy assays will be needed to assess its stability and bioavailability once it enters the bloodstream of mice, which may pose a limitation to address in future studies.

The resistance mechanisms of NET and NET/MV6 mutants were determined in strain 306, which was specifically selected for the absence of AMEs and constitutive expression of the *adeABC* efflux pump. Overexpression of the *adeABC* efflux pump is known to confer resistance to a wide range of agents, including aminoglycosides, trimethoprim, fluoroquinolones, chloramphenicol, β-lactams, erythromycin, and tetracyclines [35,48]. Other efflux pumps, such as AbeM (present in strain 306 according to WGS analysis), AbeD, and ArpAB, have also been associated with aminoglycoside resistance in *A. baumannii* [17,49,50,51]. The resistance profile of the mutants showed a reduction in susceptibility to aminoglycosides, particularly netilmicin with up to a 64-fold MIC increase, while no changes were observed with other antibiotics from the aforementioned classes, such as aztreonam or meropenem. This suggests a resistance mechanism more specific to aminoglycosides than to general *adeABC* overexpression.

Mutations identified in these spontaneous mutants included genes related to efflux pumps, specifically TetR-like regulators and ATP-binding proteins. No differences were found between NET mutants and NET/MV6 mutants, indicating that either no specific resistance mechanisms against the MV6 peptide were generated or that any existing mechanisms overlap with those for netilmicin. A hypothesis worth exploring is that MV6 and NET may compete for distinct substrate-specific binding sites within the efflux pump. Thus, a mutation causing transporter overexpression could potentially influence susceptibility to both treatments, whether used alone or in combination. Regulators from the TetR family, also referred to as TRFs, possess a helix-turn-helix (HTH) DNA-binding domain that typically enables them to function as repressors and regulates bacterial AMR [52,53]. More than 2300 nonredundant sequences belonging to this family of regulators have been identified, and it is predicted that *A. baumannii* encodes 42 of them [53,54,55]. Apart from regulating efflux pumps, such as TetA, which is associated with resistance to tetracycline-like antibiotics, other functions have also been attributed to them [56]. Examples include the gene *adeN*, which belongs to the TRF family and regulates the RND AdeIJK efflux pump [57], and *arpR*, known to regulate another RND efflux pump, ArpAB, which has been related to *A. baumannii*’s opaque/translucent colony phase variation [51]. The present mutated TetR-like regulator could potentially be involved in negatively regulating the expression of a netilmicin-related efflux pump, but its specific activity remains unstudied.

On the other hand, the ATP-binding cassette (ABC) family of transporters relies on the hydrolysis of ATP to ADP to expel substrates across the bacterial membrane [58,59]. The MacB–MacA complex is a representative member of this group, also found in *A. baumannii*, and is known to transport macrolides and gramicidin as substrates [35,60]. Moreover, a potential involvement of this ABC-type transporter in protecting *Serratia marcescens* against aminoglycosides and polymyxins has been reported [61]. Additionally, the ABC transporter MsbA requires an ATP-binding protein to export major lipids, such as LPS and phospholipids, thereby contributing to membrane integrity. The action of MV6 on altering the export of LPS, potentially affecting membrane stability, is a noteworthy area for further investigation. Strains harboring simultaneous mutations in both TetR-like regulators and ATP-binding proteins have been identified, indicating a potential synergistic effect between these alterations. In the Gram-positive *Streptomyces coelicolor* A3, sets of TRFs and adjacent ABC transport systems have been reported, where the TRFs repressed the expression of the ABC transporters within the operon [62], a relation that should be further investigated in *A. baumannii* and aminoglycosides, as it has not been documented yet.

Finally, the identification of mutations in various hypothetical proteins suggests a potential, though uncharacterized, role for these proteins in conferring netilmicin resistance. Combining computational tools such as AlphaFold and Foldseek, which can predict the functions of uncharacterized proteins, may provide valuable insights into their involvement [63]. Transcriptomic analysis and docking simulations may shed light on the specific resistance mechanism to MV6 in combination with netilmicin, strategies that are planned to be explored in the future.

### Limitations of This Study

While MV6 has demonstrated strong synergy with netilmicin, a larger and more diverse sample set encompassing various resistance profiles and mechanisms would provide deeper insights into its performance across different scenarios. Additionally, analyzing more strains that overproduce AdeABC would improve our understanding of the relationship between netilmicin resistance and MV6’s capacity to counteract the increased activity of this efflux pump. This study has also provided comprehension of the resistance mechanisms against MV6 in combination with netilmicin, which suggest the involvement of altered efflux pump expression. However, the precise mode of action could not be determined with the current experiments, highlighting the need for more specific and targeted assays in future research.

## 4. Materials and Methods

### 4.1. Molecules Used in This Study

The MV6-peptide molecules utilized in this study were commercially synthesized by GenicBio Limited (Shanghai, China). The composition and purity of MV6 were confirmed via mass spectrometry and high-performance liquid chromatography (HPLC). In addition, the reference EPI, PAβN (Sigma Aldrich, St. Louis, MO, USA), was selected to compare its activity alongside MV6. The molecular formula of PAβN is C_22_H_25_N_5_O. For experimental use, MV6 and PAβN were dissolved in pure dimethyl-sulfoxide (DMSO).

### 4.2. Strain Selection and Characterization of Aminoglycoside Modifying Enzymes (AMEs) and Relevant Efflux Pumps

A selective collection of six MDR *A. baumannii* strains with distinct antimicrobial resistance profiles was used for this study. These strains included *A. baumannii* 80, 81, 210, 306, and the colistin-resistant strain *A. baumannii* CR17 alongside its colistin-sensitive counterpart *A. baumannii* CS01 [64]. All strains are clinical isolates; strains 80, 81, 210, and 306 were isolated from patients at hospitals in a previous multicenter study [38,65]. To further characterize these strains, the presence of AMEs and relevant efflux pump genes was explored by PCR. The core cycling conditions applied were as follows: 95 °C–3 min, [94 °C–1 min; Tm–1 min; 72 °C–X min (1 min per kb)] × 35 cycles, 72 °C–45 s (final extension).

Additionally, the expression of RND-family efflux pumps (*adeABC*, *adeIJK*, and *adeFGH*) was confirmed by reverse-transcriptase real-time PCR (RT-qPCR), targeting the genes encoding the membrane transporter proteins *adeB*, *adeJ*, and *adeG*. The strains were grown overnight in LB broth, diluted 1:100 in fresh medium, and incubated at 37 °C with shaking at 180 rpm until reaching an OD600nm of 0.5. RNA was extracted using the Maxwell (R) 16 LEV simply RNA Blood Kit (Promega, Madison, WI, USA) according to the manufacturer’s instructions. To remove any potential DNA contamination, the Ambion DNA-free™ DNA Removal Kit (Thermo Fisher Scientific, Waltham, MA, USA) was used. Quality control for the RNA extractions was performed using a Nanodrop ND-1000 (Thermo Fisher), with acceptable quality parameters being a 260/280 ratio between 1.9 and 2.1 and a 260/230 ratio between 1.8 and 2.0.

For reverse transcription and cDNA generation, the PrimeScript RT Reagent Kit (Takara Bio, Kusatsu, Japan) was used following the manufacturer’ s instructions. RT-qPCR was performed using the standard protocol from Applied Biosystems™ (Fisher Scientific): 95 °C–30 s; (95 °C–15 s; 60 °C–34 s) × 45 cycles, melting curve (95 °C–15 s; 60 °C–15 s; 95 °C–15 s). Primers were designed using the Primer Express™ Software v3.0.1 from Applied Biosystems™, and different primer concentration combinations were tested to identify the most efficient conditions for the assays. *A. baumannii* ATCC 17978 was used as the reference strain for RT-qPCR quantification. Basal expression levels were controlled with *rpoB* and *gyrB* genes. Biological and technical triplicates were performed for each strain. Primer sequences and conditions are listed in Table 5.

### 4.3. Antimicrobial Susceptibility Testing

For minimum inhibitory concentration (MIC) determination, antimicrobial susceptibility testing (AST) was performed following the Clinical and Laboratory Standards Institute (CLSI) guidelines using the microdilution technique in 96-well microtiter plates. The growth medium utilized for AST was the commercial BD Phoenix™ AST Broth (Becton Dickinson, Franklin Lakes, NJ, USA) [70]. A variety of antimicrobials were evaluated, including amikacin, ceftazidime, chloramphenicol, gentamicin, levofloxacin, meropenem, netilmicin, tedizolid, and tobramycin. The MICs of MV6 alone, DMSO, and PAβN were also assessed to exclude potential interactions among these compounds in subsequent AST involving combination treatments. Preliminary studies aimed at identifying an effective concentration of MV6 for combination with antimicrobials and established that a concentration of 100 mg/L was sufficient for combined therapy. Consequently, the MV6–antibiotic combinations were tested using a fixed concentration of 100 mg/L of MV6 along with the serial dilutions of each antibiotic. Three biological replicates were conducted for each MIC determination. Subsequent studies focused on the use of NET. After AST performance, linear regression analysis was performed to assess the relationship between the *adeB* expression (relative quantification) previously mentioned and MIC values of NET alone and combined with MV6.

### 4.4. Statistical Analysis

To determine whether the expression levels of *adeB*, *adeJ*, and *adeG* differed significantly from those of the control strain *A. baumannii* ATCC 17978, a two-way analysis of variance (ANOVA) was performed. The analysis considered the RQ values as the primary factor, comparing the expression levels of these genes across different clinical strains and the control. The analysis was carried out using IBM SPSS Statistics for Windows, version 23.0 (IBM Corp., Armonk, NY, USA), following its standard procedures for two-way ANOVA. Statistical significance was set at *p*-value < 0.05. For visualization and statistical analysis of the potential correlation between NET MICs and *ade* genes expression, gene expression values were log transformed (log_10_(x + 1)) to reduce skewness and variance across samples, and AST values were log2 transformed. Data were visualized as a scatter plot with a regression line (“lm” model) superimposed onto the plot using ggplot2 package [71] (v. 3.5.1) in R [72] (v. 4.4.0). The data fit to the linear model was assessed by the coefficient of determination (R^2^), and its significance was assessed via the *p*-value using the lm function of stats package (v. 4.4.0) in R. The R^2^ value was displayed directly onto the plot for clarity.

### 4.5. Checkerboard Assays

To assess the in vitro interaction between NET and MV6, checkerboard assays were performed in 96-well microtiter plates using strains 80 and 306. Similar to MIC determination, BD Phoenix^TM^ AST Broth (Becton Dickinson, NJ, USA) medium was used for bacterial growth. A column and a row of wells were reserved for controls of each agent individually to ensure the proper concentration of the agents. Positive and negative controls were also included. Wells in rows contained serial dilutions of MV6 starting from 512 mg/L, while those in columns varied in concentrations of NET, starting at 128 mg/L and 512 mg/L for strains 306 and 80, respectively. Inoculum at ~5 × 10^5^ CFU/mL, NET, MV6, and AST broth were added to a final volume of 200 μL. Incubation followed the CLSI guidelines for MIC determination. Three biological replicates were performed. Fractional inhibitory concentration index (FICI) is defined as the summatory of FICs from compound A (MV6) and compound B (NET). FICIs were calculated and interpreted as follows:

1. FIC_MV6_ = (MIC of MV6 in combination)/(MIC of MV6 alone);
2. FIC_NET_ = (MIC of NET in combination)/(MIC of NET alone);
3. FICI = FIC_MV6_ + FIC_NET;_
4. Synergistic effect if FICI ≤ 0.5; additive effect if 0.5 < FICI ≤ 1.0; indifferent effect if 1.0 < FICI ≤ 2.0; and finally, antagonistic effect if FICI > 2.0.

### 4.6. Mutant Generation Analysis

Mutant generation was performed following a protocol adapted from Billal et al. (2007) [73]. *A. baumannii* 306 strain was selected for this process. After overnight incubation at 37 °C in Mueller–Hinton Broth (MHB), 1 × 10^8^ CFU/mL (0.5 McFarland standard) was inoculated into 2 mL of MHB supplemented with NET at concentrations ranging from 1× to 8× the MIC. In the case of combination treatments, 100 mg/L of MV6 was also added. The cultures were incubated overnight at 37 °C with shaking at 180 rpm, then plated onto Mueller–Hinton agar (MHA) plates supplemented with the corresponding concentration of NET. Ten mutants from each concentration, if any were recovered, were selected for further characterization. The selected mutants underwent susceptibility profiling using DKMGN Sensititre™ custom plates (Thermo Fisher Scientific) for Gram-negative bacteria following the manufacturer’s instructions. Two mutants from each treatment were tested for mutation reversion by incubating them on LB agar plates for 30 days with daily plating of fresh inoculum. Every 5 days, the MICs of NET and NET/MV6 were determined following the previously described procedure.

To determine the MPC, defined as the lowest antibiotic concentration that prevents the growth of resistant mutants, one-step mutants were generated [74]. Tubes containing MHB were inoculated with strain 306 and incubated for 24 h at 37 °C. The culture was then diluted to an OD_600nm_ of 0.05 and further incubated until reaching the late-exponential growth phase. Serial dilutions of *A. baumannii* 306 were plated on MHA containing varying concentrations of NET, both alone and in the presence of MV6 at 100 mg/L. In-plate concentrations of NET ranged from 1 mg/L to 1024 mg/L (Log 2 scale). Control plates without antibiotic were included to monitor the inoculum. After overnight incubation, CFUs were counted. The experiments were performed in triplicate, and the mean CFU counts were plotted to analyze mutant generation.

### 4.7. Whole-Genome Sequencing and Variant Calling

Whole-genome sequencing (WGS) was then performed on the mutants for further analysis. Ten NET/MV6 resistant mutants (one obtained at 8 mg/L, five at 16 mg/L, two at 32 mg/L, and two at 64 mg/L) and ten NET resistant mutants (five obtained at 128 mg/L and five obtained at 256 mg/L) were characterized through WGS. Genomic DNA of the mutants was extracted using the High Pure PCR Template Preparation Kit (Roche Diagnostics, Basel, Switzerland). Indexed paired-end libraries were generated using the Illumina DNA Prep library preparation kit (Illumina Inc., San Diego, CA, USA). The samples were then sequenced in a MiSeq desktop sequencer cartridge (MiSeq Reagent Kit v3, Illumina).

The variant calling analysis was performed using a pipeline developed in NextFlow DSL2. A de novo assembly, mapping, and variant identification were conducted to obtain point mutations: Single nucleotide polymorphisms (SNPs) and insertion/deletions (INDELS). De novo assembly was conducted using SPAdes (v. 3.15.3). After raw reads quality control and filtering, the “–mode novo” was followed, which performs mapping against the assembled reference strain using Bowtie2, (v. 2.2.5) [75] and SAMtools, (v. 1.14) [76]. For variant handling and identification, PicardTools, Genome Analysis Toolkit (GATK), version 4.5.0.0 [77], and FreeBayes (v. 0.9.21.7) were used after genome annotation with Prokka (v. 1.14.6) [78] and Bakta (v. 1.9.4) [79]. From the obtained VCF (variant call format) files, SNPs were listed to meet the following criteria: a quality score > 50, a root mean square (RMS) mapping quality > 25, and a coverage depth > 30. Indels were extracted from the totalpileup files using the following criteria: a quality score > 200, an RMS mapping quality > 25, and a coverage depth > 30. SNPs and INDELs for each isolate were annotated using SnpEff software (v. 4.3) [80].

## 5. Conclusions

MV6 is a cyclic peptide that lacks direct antimicrobial activity against *A. baumannii* but potentiates the activity of aminoglycosides, such as netilmicin. With a strong synergistic interaction, MV6 can reduce the MIC of netilmicin by several folds, making it a promising candidate for reversing *A. baumannii*’s resistance to this antibiotic. While the exact mode of action remains unclear, this study suggests a potential interaction with aminoglycoside efflux pumps, supported by the mutations observed in resistance mutants. These mutations are often mediated by changes in TetR-like regulators and ATP-binding proteins, both of which are involved in both the expression and activity of bacterial transporters.

## 6. Future Perspectives

*A. baumannii* is a critical pathogen, making it urgent to develop new treatment options and thoroughly investigate the resistance mechanisms that enable its extensive multidrug resistance. This study lays the foundation for further exploration of yet unknown TetR regulators and novel efflux pumps involved in aminoglycoside resistance and efflux pump expression in *A. baumannii*, underscoring the extent of what remains to be uncovered about this pathogen’s resistance mechanisms. Despite this study’s limitations, it highlights the strong relationship between aminoglycoside resistance and altered efflux pump expression. It also emphasizes how the use of adjuvant boosters, such as cyclic peptides, can help restore susceptibility, giving previously ineffective antibiotics a second chance and thereby expanding treatment options.

## Figures and Tables

**Table 1:** Results of PCR screening and the corresponding products and substrates.

Strain	Aminoglycoside Modifying Enzymes (AMEs)							Efflux Pumps				
	*aacC1*	*aacC2*	*aacA4*	*aadA1*	*aadB*	*aphA6*	*aphA1*	*adeB*	*adeJ*	*adeG*	*tetA*	*tetB*
80												
81												
210												
306												
CR17												
CS01												
Product	AAC(3)-I	AAC(3)-II	AAC(6′)-I	ANT(3″)-9	ANT(2″)-I	APH(3′)-VI	APH(3′)-I	AdeABC ^1^	AdeIJK ^1^	AdeFGH ^1^	TetA	TetB
Substrates	GM, TOB, NET	KN, NIT, GM	GM, AK, TOB	STR, SPT	GM, KN AK	AK, KN, NEO	NEO, KN	AMG, FQ, BL, CHL, TMP, TET, E, EtBr	BL, TET, FQ, CHL, TMP, FA, RIF, E, LIN, ACR, NOV, PYO, SDS	TET, TGC, NAL, FQ, SUL, EtBr, E, SDS	TET	TET, MIN
Source	[37,38,39]	[40]	[41]	[37]	[38,42]	[43]	[38]	[35]	[35]	[35]	[35]	[35]

**Color code.** Light Green: Positive PCR. White: Negative PCR. ^1^ Refers to the complete efflux pump to which the AdeB/J/G product belongs. **Abbreviations**. ACR: Acridine; AK: Amikacin; AMG: Aminoglycosides; BL: β-lactams; CHL: Chloramphenicol; E: Erythromycin; EtBr: Ethidium Bromide; FA: Fusidic Acid; FQ: Fluoroquinolones; GM: Gentamicin; KN: Kanamycin; MIN: Minocycline; NAL: Nalidixic Acid; NIT: Nitrofurantoin; NOV: Novobiocin; PYO: Pyonine; RIF: Rifampicin; SDS: Sodium Dodecyl Sulphate; SUL: Sulphonamides; SPT: Spectinomycin; STR: Streptomycin; TET: Tetracycline; TGC: Tigecycline; TMP: Trimethoprim; TOB: Tobramycin.

**Table 2:** Minimum inhibitory concentrations (MICs) in mg/L of NET, alone or in combination with PAβN and MV6, for selected *A. baumannii* strains. Both PAβN and MV6 are used at a constant concentration of 100 mg/L.

Strains *A. baumannii*	NET	NET + MV6	Fold-Change	NET + PAβN	Fold-Change
80	256	32	8-fold	128	2-fold
81	256	32	8-fold	64	4-fold
210	16	8 *	2-fold	8 *	2-fold
306	64	8 *	8-fold	16	4-fold
CR17	32	8 *	4-fold	8 *	4-fold
CS01	32	8 *	4-fold	16	2-fold

* MIC that breaks the CLSI resistance breakpoint of NET (≥16 mg/L) for *A. baumannii*.

**Table 3:** Variation in the resistance profile of the NET and NET/MV6 spontaneous mutants compared to the original parental strain *A. baumannii* 306.

		MIC (mg/L)		Sensititre (DKNGM)																
Mutant Strains		[NET]	[NET]/MV6	MERO	GEN	CIP	AUGC	COL	TGC	TAZ	IMI	AZT	C/T	SXT	P/T4	FOT	CZA	ETP	AMI	TOB
AB1	WT 306	64	8	16	8	>2	>64/2	1	2	>16	>16	32	16/4	>8/152	>32/4	>8	>16/4	>2	8	2
AB2	Ab306_NET8_MV6	1024	32	16	>8	>2	>64/2	1	1	>16	>16	16	16/4	>8/152	>32/4	>8	>16/4	>2	16	2
AB3	Ab306_NET32_MV6	512	64	16	>8	>2	>64/2	1	1	>16	>16	32	>32/4	>8/152	>32/4	>8	>16/4	>2	32	2
AB4	Ab306_NET64_MV6	4096	512	16	>8	>2	>64/2	0.5	>4	>16	16	16	32/4	>8/152	>32/4	>8	>16/4	>2	32	8
AB5	Ab306_NET64_MV6	4096	512	8	>8	>2	>64/2	0.5	4	>16	>16	16	32/4	>8/152	>32/4	>8	>16/4	>2	32	8
AB6	Ab306_NET32_MV6	2048	512	>16	>8	>2	>64/2	0.5	>4	>16	>16	16	>32/4	>8/152	>32/4	>8	>16/4	>2	16	8
AB7	Ab306_NET16_MV6	1024	128	16	>8	>2	>64/2	0.5	>4	>16	16	16	16/4	>8/152	>32/4	>8	>16/4	>2	16	8
AB8	Ab306_NET16_MV6	512	256	8	>8	>2	>64/2	0.5	4	>16	>16	32	32/4	>8/152	>32/4	>8	>16/4	>2	16	8
AB9	Ab306_NET16_MV6	512	256	16	>8	>2	>64/2	0.5	4	>16	16	16	32/4	>8/152	>32/4	>8	>16/4	>2	32	8
AB10	Ab306_NET16_MV6	512	256	16	>8	>2	>64/2	0.5	4	>16	16	8	32/4	>8/152	>32/4	>8	>16/4	>2	32	8
AB11	Ab306_NET16_MV6	512	256	16	>8	>2	>64/2	0.5	4	>16	16	8	16/4	>8/152	>32/4	>8	>16/4	>2	32	8
AB12	Ab306_NET128	512	256	16	>8	>2	>64/2	1	2	>16	>16	16	32/4	>8/152	>32/4	>8	>16/4	>2	8	2
AB13	Ab306_NET256	1024	128	16	>8	>2	>64/2	1	4	>16	>16	16	16/4	>8/152	>32/4	>8	>16/4	>2	8	4
AB14	Ab306_NET256	2048	512	>16	>8	>2	>64/2	1	>4	>16	>16	32	32/4	>8/152	>32/4	>8	>16/4	>2	32	8
AB15	Ab306_NET256	2048	256	16	>8	>2	>64/2	0.5	4	>16	>16	16	32/4	>8/152	>32/4	>8	>16/4	>2	16	8
AB16	Ab306_NET256	2048	256	16	>8	>2	>64/2	0.5	4	>16	>16	8	32/4	>8/152	>32/4	>8	>16/4	>2	32	8
AB17	Ab306_NET256	4096	512	16	>8	>2	>64/2	0.5	4	>16	>16	16	32/4	>8/152	>32/4	>8	>16/4	>2	16	8
AB18	Ab306_NET128	2048	256	16	>8	>2	>64/2	1	4	>16	>16	16	32/4	>8/152	>32/4	>8	>16/4	>2	32	>8
AB19	Ab306_NET128	2048	256	16	>8	>2	>64/2	0.5	>4	>16	>16	16	32/4	>8/152	>32/4	>8	>16/4	>2	16	8
AB20	Ab306_NET128	4096	256	>16	>8	>2	>64/2	0.5	4	>16	>16	32	32/4	>8/152	>32/4	>8	>16/4	>2	32	8
AB21	Ab306_NET128	2048	256	16	>8	>2	>64/2	0.5	4	>16	>16	32	>32/4	>8/152	>32/4	>8	>16/4	>2	32	8

**Examples of mutant strain nomenclature.** Ab306_NET8_MV6: derivative mutant from *A. baumannii* 306, isolated at NET 8 mg/L in the presence of 100 mg/L of MV6. Ab306_NET128: derivative mutant from *A. baumannii* 306, isolated at NET 128 mg/L, monotherapy. **Abbreviations**. NET: Netilmicin; MERO: Meropenem; GEN: Gentamicin; CIP: Ciprofloxacin; AUGC: Amoxicillin/clavulanic acid constant 2; COL: Colistin; TGC: Tigecycline; TAZ: Ceftazidime; IMI: Imipenem; AZT: Aztreonam; C/T: Ceftolozane/tazobactam 4; SXT: Trimethoprim/sulfamethoxazole; P/T4: Piperacillin/tazobactam constant 4; FOT: Cefotaxime; CZA: Ceftazidime/Avivactam; ETP: Ertapenem; AMI: Amikacin; TOB: Tobramycin.

**Table 4:** Variant calling analysis of spontaneous mutants of NET and netilmicin with MV6 (NET/MV6).

**NET/MV6**						
**Mutant**	**Gene**	**Mutation**	**Annotation (NCBI)**	**Reference (NCBI)**	**Impact**	**Type**
AB2	ATP-binding protein	SNP 865 G>A/A289T	GBFHJJIP_03385	WP_001207474.1	MOD	MV
	Intergenic TetR/AcrR family	SNP (T>C)	GBFHJJIP_02069_gene-CHR_END	GBFHJJIP_02069	M/L	IR
AB3	ATP-binding protein	SNP 865 G>A/A289T	GBFHJJIP_03385	WP_001207474.1	MOD	MV
AB4	ATP-binding protein	SNP 865 G>A/A289T	GBFHJJIP_03385	WP_001207474.1	MOD	MV
	*adeR*	DEL 40274 (TCTCCACACTTA>T)	GBFHJJIP_02896_gene/GBFHJJIP_02897_gene	WP_000459542.1	M/L	IR
AB5	**TetR family**	INS 363 (C>CAT)	GBFHJJIP_01215	GBFHJJIP_01215	**HIGH**	FV
	ATP-binding protein	SNP 865 G>A/A289T	GBFHJJIP_03385	WP_001207474.1	MOD	MV
AB6	**TetR family**	DEL 364 (182 nucleotides>C)	GBFHJJIP_01215	GBFHJJIP_01215	**HIGH**	FV
	HP [domain cpo]	INS 155 (T>TGGACGTGGA)	GBFHJJIP_03384	HMPREF0010_00495	MOD	DII
	ATP-binding protein	SNP 865 G>A/A289T	GBFHJJIP_03385	WP_001207474.1	MOD	MV
AB7	ATP-binding protein	SNP 1069 A>C/T357P SNP 1086 A>C/E362D	GBFHJJIP_03385	WP_001207474.1	MOD	MV
AB8	HP [domain fadD]	SNP 192 T>G/H64Q	GBFHJJIP_03655	D0CB89_ACIB2	MOD	MV
AB9	*adeR*	DEL (TCTCCACACTTA>T)	GBFHJJIP_02896_gene-GBFHJJIP_02897_gene	WP_000459542.1	M/L	IR
	TetR/AcrR family	SNP (T>C)	GBFHJJIP_02069_gene-CHR_END	GBFHJJIP_02069	M/L	IR
AB10	ATP-binding protein	SNP 1069 A>C/T357P SNP 1086 A>C/E362D	GBFHJJIP_03385	WP_001207474.1	MOD	MV
AB11	HP [domain fadD]	SNP 192 T>G/H64Q	GBFHJJIP_03655	D0CB89_ACIB2	MOD	MV
	HP [domain GntR family]	SNP 197 T>C/I66T	GBFHJJIP_03660	HMPREF0010_00945	MOD	MV
**NET**						
**Mutant**	**Gene**	**Mutation**	**Annotation (NCBI)**	**Reference (NCBI)**	**Impact**	**Type**
AB12	ATP-binding protein	SNP 865 G>A/A289T	GBFHJJIP_03385	WP_001207474.1	MOD	MV
AB13	ATP-binding protein	SNP 865 G>A/A289T	GBFHJJIP_03385	WP_001207474.1	MOD	MV
AB14	*adeR*–*adeA*	DEL (TCTCCACACTTA>T)	GBFHJJIP_02896_gene-GBFHJJIP_02897_gene	WP_000459542.1	M/L	IR
	TetR/AcrR family	SNP (A>G)	GBFHJJIP_02069_gene y CHR_END	GBFHJJIP_02069	M/L	IR
AB15	**TetR family**	INS 512 (T>20 nucleotides)	GBFHJJIP_01215	GBFHJJIP_01215	**HIGH**	**FV/S**
	HP [domain fadD]	SNP 192 T>G/H64Q	GBFHJJIP_03655	D0CB89_ACIB2	MOD	MV
	HP [domain GntR family]	SNP 197 T>C/I66T	GBFHJJIP_03660	HMPREF0010_00945	MOD	MV
AB16	**TetR family**	DEL 174 (18 nucleotides>A)	GBFHJJIP_01215	GBFHJJIP_01215	**HIGH**	**FV**
	ATP-binding protein	SNP 865 G>A/A289T	GBFHJJIP_03385	WP_001207474.1	MOD	MV
AB17	ATP-binding protein	SNP 1069 A>C/T357P	GBFHJJIP_03385	WP_001207474.1	MOD	MV
	HP [domain GntR family]	SNP 197 T>C/I66T	GBFHJJIP_03660	HMPREF0010_00945	MOD	MV
	**TetR family**	INS 511 (T>TCTG)	GBFHJJIP_01215	GBFHJJIP_01215	**HIGH**	**DII**
AB18	*adeR*–*adeA*	DEL (TCTCCACACTTA>T)	GBFHJJIP_02896_gene-GBFHJJIP_02897_gene	WP_000459542.1	M/L	IR
	TetR/AcrR family	SNP (A>G)	GBFHJJIP_02069	GBFHJJIP_02069	M/L	IR
AB19	ATP-binding protein	SNP 1069 A>C/T357P	GBFHJJIP_03385	WP_001207474.1	MOD	MV
	HP [domain fadD]	SNP 192 T>G/H64Q	GBFHJJIP_03655	D0CB89_ACIB2	MOD	MV
AB20	HP [domain fadD]	SNP 192 T>G/H64Q	GBFHJJIP_03655	D0CB89_ACIB2	MOD	MV
	TetR/AcrR family	SNP (A>G)	GBFHJJIP_02069	GBFHJJIP_02069	M/L	IR
	*adeR*–*adeA*	DEL (TCTCCACACTTA>T)	GBFHJJIP_02896_gene-GBFHJJIP_02897_gene	WP_000459542.1	M/L	IR
	HP [domain fadD]	SNP (A>G)	CHR_START/GBFHJJIP_03655	GBFHJJIP_03655	M/L	IR
AB21	TetR/AcrR family	SNP (T>C)	GBFHJJIP_02069	GBFHJJIP_02069	M/L	IR
	HP [domain fadD]	SNP (A>C)	GBFHJJIP_03655	D0CB89_ACIB2	M/L	IR
	PA4642 family protein/tyrosyl-tRNA synthetase	INS (>CAATCAAATCA)	GBFHJJIP_01042_gene/GBFHJJIP_01043_gene	WP_001218560.1–WP_031969348.1	M/L	IR
	*adeR*–*adeA*	DEL (TCTCCACACTTA>)	GBFHJJIP_02896_gene-GBFHJJIP_02897_gene	WP_000459542.1	M/L	IR

**Column gene.** HP: Hypothetical protein. **Column Mutation.** SNP: Single Nucleotide Polymorphism; DEL: Deletion; INS: Insertion. **Column impact.** MOD: Moderate; M/L: Modifier/Low. **Column Type.** MV: Missense variant; IR: Intergenic Region; FV: Frameshift Variant; S: Stop gained; DII: Disruptive In-frame Insertion. Mutations with a prediction of High Impact are marked in bold.

**Table 5:** PCR primers used in this study.

Gene	Primer Sequence (5′–3′)	Tm (°C)	Length (bp)	Source
*aacC1*	1: ATGGGCATCATTCGCACATGTAGG	52	456	[66]
	2: TTAGGTGGCGGTACTTGGGTC			
*aacC2*	1. ATTGATTCAGCAGGCCGAAC	59	247	[67]
	2: CTCTTGATGGTGCATGCCTC			
*aacA4*	1: TTGCGATGCTCTATGAGTGGCTA	63	482	[68]
	2: CTCGAATGCCTGGCGTGTTT			
*aadA1*	1: ATGAGGGAAGCGGTGATCG	52	792	[66]
	2: TTATTTGCCGACTACCTTGGTG			
*aadB*	1: ATGGACACAACGCAGGTCGC	55	534	[66]
	2: TTAGGCCGCATATCGCGACC			
*aphA6*	1: ATGGAATTGCCCAATATTATTC	55	797	[66]
	2: TCAATTCAATTCATCAAGTTTTA			
*aphA1*	1: AAACGTCTTGCTCGAGGC	56	461	[68]
	2: CAAACCGTTATTCATTCGTGA			
*adeB*	1: ATGTCACAATTTTTTATTCGTCGTC	56	3104	[69]
	2: TTAGGATGAGATTTTTTTCTTAGAGG			
*adeJ*	1: CTGGCTTATGACACGACTC	61	988	[69]
	2: GGATCCCCATACCACGCTGG			
*adeG*	1: GTTGCTCGTGTCGAACTTGC	57	918	[69]
	2: AGGAACGAAACCACCTGGAAC			
*tetA*	1: GTAATTCTGAGGACTGTCGC	55	950	[16]
	2: CTGCCTGGACAACATTGCTT			
*tetB*	1: TTGGTTAGGGGCAAGTTTTG	56	659	[16]
	2: GTAATGGGCCAATAACACCG			
*adeB* (RT-qPCR)	1: CTGCTGTACCGGAGGTATCTGTT	60	~60	This study
	2: GCGCGAATTATCGGGTGTAA			
*adeJ* (RT-qPCR)	1: AGGCGAATGGACGTATGGTT	60	~60	This study
	2: AACCGATGACACGCCGTTA			
*adeG* (RT-qPCR)	1: CGCGACCGAAATTGTGAAT	60	~60	This study
	2: GATTGTACCCGCTGCAACCT			
*gyrB* (RT-qPCR)	1: CTGCAGCAGAAACCCCTTCT	60	~60	This study
	2: ATAATGGCCGCGGTATTCC			
*rpoB* (RT-qPCR)	1: TCCATTCCTTGAACACGATGAC	60	~60	This study
	2: CTGCCTGACGTTGCATGTTT			

Tm: annealing temperature. bp: base pairs.

## Data Availability

The data presented from the whole-genome sequencing analysis in this study are openly available on GitHub “https://github.com/AMRmicrobiology/WGS-Analysis-VariantCalling (accessed on 14 November 2024)”. The rest of the data presented in this study are available upon request from the corresponding author.

## References

[1] Peleg A.Y., Harald S., Paterson D.L. (2008). *Acinetobacter baumannii*: Emergence of a Successful Pathogen. Clin. Microbiol. Rev..

[2] Poirel L., Nordmann P. (2006). Carbapenem Resistance in *Acinetobacter baumannii*: Mechanisms and Epidemiology. Clin. Microbiol. Infect..

[3] Vaneechoutte M., Young D.M., Ornston L.N., De Baere T., Nemec A., Van Der Reijden T., Carr E., Tjernberg I., Dijkshoorn L. (2006). Naturally Transformable Acinetobacter Sp. Strain ADP1 Belongs to the Newly Described Species Acinetobacter Baylyi. Appl. Environ. Microbiol..

[4] Fournier P.-E., Vallenet D., Barbe V., Audic S., Ogata H., Poirel L., Richet H., Robert C., Mangenot S., Abergel C. (2006). Comparative Genomics of Multidrug Resistance in *Acinetobacter baumannii*. PLoS Genet..

[5] WHO (2024). WHO Bacterial Priority Pathogens List, 2024.

[6] Murray C.J., Ikuta K.S., Sharara F., Swetschinski L., Robles Aguilar G., Gray A., Han C., Bisignano C., Rao P., Wool E. (2022). Global Burden of Bacterial Antimicrobial Resistance in 2019: A Systematic Analysis. Lancet.

[7] Ma C., McClean S. (2021). Mapping Global Prevalence of *Acinetobacter baumannii* and Recent Vaccine Development to Tackle It. Vaccines.

[8] McConnell M.J., Actis L., Pachón J. (2013). *Acinetobacter baumannii*: Human Infections, Factors Contributing to Pathogenesis and Animal Models. FEMS Microbiol. Rev..

[9] Antunes L.C.S., Visca P., Towner K.J. (2014). *Acinetobacter baumannii*: Evolution of a Global Pathogen. Pathog. Dis..

[10] Roca I., Espinal P., Vila-farrés X., Vila J. (2012). The *Acinetobacter baumannii* Oxymoron: Commensal Hospital Dweller Turned Pan-Drug-Resistant Menace. Front. Microbiol..

[11] Wieczorek P., Sacha P., Hauschild T., Zórawski M., Krawczyk M., Tryniszewska E. (2008). Multidrug Resistant *Acinetobacter baumannii*—The Role of AdeABC (RND Family) Efflux Pump in Resistance to Antibiotics. Folia Histochem. Cytobiol..

[12] Xu C.F., Bilya S.R., Xu W. (2019). AdeABC Efflux Gene in *Acinetobacter baumannii*. New Microbes New Infect..

[13] Damier-Piolle L., Magnet S., Brémont S., Lambert T., Courvalin P. (2008). AdeIJK, a Resistance-Nodulation-Cell Division Pump Effluxing Multiple Antibiotics in *Acinetobacter baumannii*. Antimicrob. Agents Chemother..

[14] Leus I.V., Weeks J.W., Bonifay V., Smith L., Richardson S., Zgurskaya H.I. (2018). Substrate Specificities and Efflux Efficiencies of RND Efflux Pumps of *Acinetobacter baumannii*. J. Bacteriol..

[15] Roca I., Marti S., Espinal P., Martínez P., Gibert I., Vila J. (2009). CraA, a Major Facilitator Superfamily Efflux Pump Associated with Chloramphenicol Resistance in *Acinetobacter baumannii*. Antimicrob. Agents Chemother..

[16] Martí S., Fernández-Cuenca F., Pascual Á., Ribera A., Rodríguez-Baño J., Bou G., Miguel Cisneros J., Pachón J., Martínez-Martínez L., Vila J. (2006). Prevalencia de Los Genes TetA y TetB Como Mecanismo de Resistencia a Tetraciclina y Minociclina En Aislamientos Clínicos de *Acinetobacter baumannii*. Enferm. Infecc. Microbiol. Clin..

[17] Xian-Zhong S., Jing C., Tohru M., Teruo K., Tomofusa T. (2005). AbeM, an H+-Coupled *Acinetobacter baumannii* Multidrug Efflux Pump Belonging to the MATE Family of Transporters. Antimicrob. Agents Chemother..

[18] Couvé-Deacon E., Jové T., Afouda P., Barraud O., Tilloy V., Scaon E., Hervé B., Burucoa C., Kempf M., Marcos J.Y. (2019). Class 1 Integrons in *Acinetobacter baumannii*: A Weak Expression of Gene Cassettes to Counterbalance the Lack of LexA-Driven Integrase Repression. Int. J. Antimicrob. Agents.

[19] Butler M.S., Gigante V., Sati H., Paulin S., Al-Sulaiman L., Rex J.H., Fernandes P., Arias C.A., Paul M., Thwaites G.E. (2022). Analysis of the Clinical Pipeline of Treatments for Drug-Resistant Bacterial Infections: Despite Progress, More Action Is Needed. Antimicrob. Agents Chemother..

[20] Hesterkamp T., Stadler M., Dersch P. (2016). Antibiotics Clinical Development and Pipeline BT—How to Overcome the Antibiotic Crisis: Facts, Challenges, Technologies and Future Perspectives.

[21] Provenzani A., Hospodar A.R., Meyer A.L., Leonardi Vinci D., Hwang E.Y., Butrus C.M., Polidori P. (2020). Multidrug-Resistant Gram-Negative Organisms: A Review of Recently Approved Antibiotics and Novel Pipeline Agents. Int. J. Clin. Pharm..

[22] Gatti M., Cosentino F., Giannella M., Viale P., Pea F. (2024). Clinical Efficacy of Cefiderocol-Based Regimens in Patients with Carbapenem-Resistant *Acinetobacter baumannii* Infections: A Systematic Review with Meta-Analysis. Int. J. Antimicrob. Agents.

[23] Scott C.J., Zhu E., Jayakumar R.A., Shan G., Viswesh V. (2022). Efficacy of Eravacycline Versus Best Previously Available Therapy for Adults With Pneumonia Due to Difficult-to-Treat Resistant (DTR) *Acinetobacter baumannii*. Ann. Pharmacother..

[24] Alosaimy S., Morrisette T., Lagnf A.M., Rojas L.M., King M.A., Pullinger B.M., Hobbs A.L.V., Perkins N.B., Veve M.P., Bouchard J. (2022). Clinical Outcomes of Eravacycline in Patients Treated Predominately for Carbapenem-Resistant *Acinetobacter baumannii*. Microbiol. Spectr..

[25] Landman D., Kelly P., Bäcker M., Babu E., Shah N., Bratu S., Quale J. (2011). Antimicrobial Activity of a Novel Aminoglycoside, ACHN-490, against *Acinetobacter baumannii* and *Pseudomonas aeruginosa* from New York City. J. Antimicrob. Chemother..

[26] Keam S.J. (2023). Sulbactam/Durlobactam: First Approval. Drugs.

[27] Nguyen L.P., Pinto N.A., Vu T.N., Lee H., Cho Y.L., Byun J.-H., D’Souza R., Yong D. (2020). In Vitro Activity of a Novel Siderophore-Cephalosporin, GT-1 and Serine-Type β-Lactamase Inhibitor, GT-055, against *Escherichia coli*, *Klebsiella pneumoniae* and *Acinetobacter* spp. Panel Strains. Antibiotics.

[28] Saito H., Yoshikuni O., Megumi C., Kazuki H., Naomasa G. (2013). Potent In Vitro Antibacterial Activity of DS-8587, a Novel Broad-Spectrum Quinolone, against *Acinetobacter baumannii*. Antimicrob. Agents Chemother..

[29] Isler B., Doi Y., Bonomo R.A., Paterson D.L. (2018). New Treatment Options against Carbapenem-Resistant *Acinetobacter baumannii* Infections. Antimicrob. Agents Chemother..

[30] Awan R.E., Zainab S., Yousuf F.J., Mughal S. (2024). AI-Driven Drug Discovery: Exploring Abaucin as a Promising Treatment against Multidrug-Resistant *Acinetobacter baumannii*. Health Sci. Rep..

[31] Hua Y., Luo T., Yang Y., Dong D., Wang R., Wang Y., Xu M., Guo X., Hu F., He P. (2018). Phage Therapy as a Promising New Treatment for Lung Infection Caused by Carbapenem-Resistant *Acinetobacter baumannii* in Mice. Front. Microbiol..

[32] Lood R., Winer B.Y., Pelzek A.J., Diez-Martinez R., Thandar M., Euler C.W., Schuch R., Fischetti V.A. (2015). Novel Phage Lysin Capable of Killing the Multidrug-Resistant Gram-Negative Bacterium *Acinetobacter baumannii* in a Mouse Bacteremia Model. Antimicrob. Agents Chemother..

[33] Nielsen T.B., Pantapalangkoor P., Luna B.M., Bruhn K.W., Yan J., Dekitani K., Hsieh S., Yeshoua B., Pascual B., Vinogradov E. (2017). Monoclonal Antibody Protects Against *Acinetobacter baumannii* Infection by Enhancing Bacterial Clearance and Evading Sepsis. J. Infect. Dis..

[34] McConnell M.J., Rumbo C., Bou G., Pachón J. (2011). Outer Membrane Vesicles as an Acellular Vaccine against *Acinetobacter baumannii*. Vaccine.

[35] Verma P., Tiwari M., Tiwari V. (2021). Efflux Pumps in Multidrug-Resistant *Acinetobacter baumannii*: Current Status and Challenges in the Discovery of Efflux Pumps Inhibitors. Microb. Pathog..

[36] Xing L., Barnie P.A., Su Z., Xu H. (2014). Development of Efflux Pumps and Inhibitors (EPIs) in *A. baumanii*. Clin. Microb..

[37] Post V., Hall R.M. (2009). AbaR5, a Large Multiple-Antibiotic Resistance Region Found in *Acinetobacter baumannii*. Antimicrob. Agents Chemother..

[38] Rumbo C., Gato E., López M., Ruiz de Alegría C., Fernández-Cuenca F., Martínez-Martínez L., Vila J., Pachón J., Cisneros J.M., Rodríguez-Baño J. (2013). Contribution of Efflux Pumps, Porins, and β-Lactamases to Multidrug Resistance in Clinical Isolates of *Acinetobacter baumannii*. Antimicrob. Agents Chemother..

[39] Shi W.F., Jiang J.P., Ml Z.H. (2024). Relationship between Antimicrobial Resistance and Aminoglycoside-Modifying Enzyme Gene Expressions in *Acinetobacter baumannii*. Chin. Med. J..

[40] Onohuean H., Nwodo U.U. (2023). Polymorphism and Mutational Diversity of Virulence (VcgCPI/VcgCPE) and Resistance Determinants (Aac(3)-IIa, (AacC2, StrA, Sul 1, and 11) among Human Pathogenic Vibrio Species Recovered from Surface Waters in South-Western Districts of Uganda. J. Genet. Eng. Biotechnol..

[41] Sacha P., Jaworowska J., Ojdana D., Wieczorek P., Czaban S., Tryniszewska E. (2012). Occurrence of the *AacA4* Gene among Multidrug Resistant Strains of *Pseudomonas aeruginosa* Isolated from Bronchial Secretions Obtained from the Intensive Therapy Unit at University Hospital in Bialystok, Poland. Folia Histochem. Cytobiol..

[42] Cabrera R., Fernández-Barat L., Vázquez N., Alcaraz-Serrano V., Bueno-Freire L., Amaro R., López-Aladid R., Oscanoa P., Muñoz L., Vila J. (2022). Resistance Mechanisms and Molecular Epidemiology of *Pseudomonas aeruginosa* Strains from Patients with Bronchiectasis. J. Antimicrob. Chemother..

[43] Huang F.C., Klaus S., Herz S., Zou Z., Koop H.U., Golds T. (2002). Efficient Plastid Transformation in Tobacco Using the AphA-6 Gene and Kanamycin Selection. Mol. Genet. Genom..

[44] Neshani A., Sedighian H., Mirhosseini S.A., Ghazvini K., Zare H., Jahangiri A. (2020). Antimicrobial Peptides as a Promising Treatment Option against *Acinetobacter baumannii* Infections. Microb. Pathog..

[45] Rangel K., Lechuga G.C., Provance D.W., Morel C.M., De Simone S.G. (2023). An Update on the Therapeutic Potential of Antimicrobial Peptides against *Acinetobacter baumannii* Infections. Pharmaceuticals.

[46] Panjla A., Kaul G., Chopra S., Titz A., Verma S. (2021). Short Peptides and Their Mimetics as Potent Antibacterial Agents and Antibiotic Adjuvants. ACS Chem. Biol..

[47] Chen C., Shi J., Wang D., Kong P., Wang Z., Liu Y. (2024). Antimicrobial Peptides as Promising Antibiotic Adjuvants to Combat Drug-Resistant Pathogens. Crit. Rev. Microbiol..

[48] Magnet S., Courvalin P., Lambert T. (2001). Resistance-Nodulation-Cell Division-Type Efflux Pump Involved in Aminoglycoside Resistance in *Acinetobacter baumannii* Strain BM4454. Antimicrob. Agents Chemother..

[49] Srinivasan V.B., Venkataramaiah M., Mondal A., Rajamohan G. (2015). Functional Characterization of AbeD, an RND-Type Membrane Transporter in Antimicrobial Resistance in *Acinetobacter baumannii*. PLoS ONE.

[50] Tipton K.A., Farokhyfar M., Rather P.N. (2017). Multiple Roles for a Novel RND Type Efflux System in *Acinetobacter baumannii* AB5075. Microbiologyopen.

[51] Kornelsen V., Kumar A. (2021). Update on Multidrug Resistance Efflux Pumps in *Acinetobacter* spp.. Antimicrob. Agents Chemother..

[52] Cuthbertson L., Nodwell J.R. (2013). The TetR Family of Regulators. Microbiol. Mol. Biol. Rev..

[53] Ramos J.L., Martínez-Bueno M., Molina-Henares A.J., Terán W., Watanabe K., Zhang X., Gallegos M.T., Brennan R., Tobes R. (2005). The TetR Family of Transcriptional Repressors. Microbiol. Mol. Biol. Rev..

[54] Martínez-Bueno M., Molina-Henares A.J., Pareja E., Ramos J.L., Tobes R. (2004). BacTregulators: A Database of Transcriptional Regulators in Bacteria and Archaea. Bioinformatics.

[55] Casella L.G., Weiss A., Pérez-Rueda E., Antonio Ibarra J., Shaw L.N. (2017). Towards the Complete Proteinaceous Regulome of *Acinetobacter baumannii*. Microb. Genom..

[56] Beck C.F., Mutzel R., Barbé J., Müller W. (1982). A Multifunctional Gene (TetR) Controls Tn10-Encoded Tetracycline Resistance. J. Bacteriol..

[57] Rosenfeld N., Bouchier C., Courvalin P., Périchon B. (2012). Expression of the Resistance-Nodulation-Cell Division Pump AdeIJK in *Acinetobacter baumannii* Is Regulated by AdeN, a TetR-Type Regulator. Antimicrob. Agents Chemother..

[58] Abdi S.N., Ghotaslou R., Ganbarov K., Mobed A., Tanomand A., Yousefi M., Asgharzadeh M., Kafil H.S. (2020). *Acinetobacter baumannii* Efflux Pumps and Antibiotic Resistance. Infect. Drug Resist..

[59] De Gaetano G.V., Lentini G., Famà A., Coppolino F., Beninati C. (2023). Antimicrobial Resistance: Two-Component Regulatory Systems and Multidrug Efflux Pumps. Antibiotics.

[60] Okada U., Yamashita E., Neuberger A., Morimoto M., van Veen H.W., Murakami S. (2017). Crystal Structure of Tripartite-Type ABC Transporter MacB from *Acinetobacter baumannii*. Nat. Commun..

[61] Shirshikova T.V., Sierra-Bakhshi C.G., Kamaletdinova L.K., Matrosova L.E., Khabipova N.N., Evtugyn V.G., Khilyas I.V., Danilova I.V., Mardanova A.M., Sharipova M.R. (2021). The ABC-Type Efflux Pump MacAB Is Involved in Protection of Serratia Marcescens against Aminoglycoside Antibiotics, Polymyxins, and Oxidative Stress. mSphere.

[62] Yukun L., Shumpei A., Takumi I., Hiroyasu O. (2023). Regulation of Multidrug Efflux Pumps by TetR Family Transcriptional Repressor Negatively Affects Secondary Metabolism in Streptomyces Coelicolor A3(2). Appl. Environ. Microbiol..

[63] Hutson M. (2023). Foldseek Gives AlphaFold Protein Database a Rapid Search Tool. Nature.

[64] López-Rojas R., Jiménez-Mejías M.E., Lepe J.A., Pachón J. (2011). *Acinetobacter baumannii* Resistant to Colistin Alters Its Antibiotic Resistance Profile: A Case Report From Spain. J. Infect. Dis..

[65] Fernández-Cuenca F., Tomás-carmona M., Caballero-moyano F., Bou G., Martínez-martínez L., Vila J., Pachón J., Miguel J. (2013). Actividad de 18 Agentes Antimicrobianos Frente a Aislados Clínicos de *Acinetobacter baumannii*: Segundo Estudio Nacional Multicéntrico (Proyecto GEIH-REIPI-Ab 2010). Enfermedades Infecc. Microbiol. Clin..

[66] Kishk R., Soliman N., Nemr N., Eldesouki R., Mahrous N., Gobouri A., Azab E., Anani M. (2021). Prevalence of Aminoglycoside Resistance and Aminoglycoside Modifying Enzymes in *Acinetobacter baumannii* Among Intensive Care Unit Patients, Ismailia, Egypt. Infect. Drug Resist..

[67] Chegeni F.B., Cheraghipour K., Shakib P. (2020). Detection of Aacc1 and Aacc2 Genes in Clinical Isolates of Klebsiella Pneumoniae. Int. J. Med. Investig..

[68] Rastegar Lari A., Valadan Tahbaz S. (2019). Characterization of aminoglycoside resistance mechanisms in *Acinetobacter baumannii* isolates from burn wound colonization mécanismes de résistance aux aminosides *d’Acinetobacter baumannii* isolé de zones brûlées. Ann. Fires Burn Disaster.

[69] Cosgaya C., Ratia C., Marí-Almirall M., Rubio L., Higgins P.G., Seifert H., Roca I., Vila J. (2019). In Vitro and in Vivo Virulence Potential of the Emergent Species of the *Acinetobacter baumannii* (Ab) Group. Front. Microbiol..

[70] CLSI (2020). M100 Performance Standards for Antimicrobial Susceptibility Testing.

[71] Wickham H., Wickham H. (2016). Data Transformation BT—Ggplot2: Elegant Graphics for Data Analysis.

[72] R Core Team (2024). R: A Language and Environment for Statistical Computing.

[73] Billal D.S., Fedorko D.P., Yan S.S., Hotomi M., Fujihara K., Nelson N., Yamanaka N. (2007). In Vitro Induction and Selection of Fluoroquinolone-Resistant Mutants of Streptococcus Pyogenes Strains with Multiple Emm Types. J. Antimicrob. Chemother..

[74] Drlica K. (2003). The Mutant Selection Window and Antimicrobial Resistance. J. Antimicrob. Chemother..

[75] Langmead B., Salzberg S.L. (2012). Fast Gapped-Read Alignment with Bowtie 2. Nat. Methods.

[76] Danecek P., Bonfield J.K., Liddle J., Marshall J., Ohan V., Pollard M.O., Whitwham A., Keane T., McCarthy S.A., Davies R.M. (2021). Twelve Years of SAMtools and BCFtools. Gigascience.

[77] McKenna A., Hanna M., Banks E., Sivachenko A., Cibulskis K., Kernytsky A., Garimella K., Altshuler D., Gabriel S., Daly M. (2010). The Genome Analysis Toolkit: A MapReduce Framework for Analyzing next-Generation DNA Sequencing Data. Genome Res..

[78] Seemann T. (2014). Prokka: Rapid Prokaryotic Genome Annotation. Bioinformatics.

[79] Schwengers O., Jelonek L., Dieckmann M.A., Beyvers S., Blom J., Goesmann A. (2021). Bakta: Rapid and Standardized Annotation of Bacterial Genomes via Alignment-Free Sequence Identification. Microb. Genom..

[80] Cingolani P., Platts A., Wang L.L., Coon M., Nguyen T., Wang L., Land S.J., Lu X., Ruden D.M. (2012). A Program for Annotating and Predicting the Effects of Single Nucleotide Polymorphisms, SnpEff: SNPs in the Genome of Drosophila Melanogaster Strain W1118; Iso-2; Iso-3. Fly.

