# Correction: Roson-Calero et al. Cyclic Peptide MV6, an Aminoglycoside Efficacy Enhancer Against *Acinetobacter baumannii*. *Antibiotics* 2024, *13*, 1147

**DOI:** 10.3390/antibiotics14020174

**Published:** 2025-02-11

**Authors:** Natalia Roson-Calero, Jimmy Lucas, María A. Gomis-Font, Roger de Pedro-Jové, Antonio Oliver, Clara Ballesté-Delpierre, Jordi Vila

**Affiliations:** 1 Barcelona Institute for Global Health (ISGlobal), 08036 Barcelona, Spainjimmy.lucas@isglobal.org (J.L.);; 2 Department of Basic Clinical Practice, School of Medicine, University of Barcelona, 08036 Barcelona, Spain; 3 CIBER de Enfermedades Infecciosas (CIBERINFEC), Instituto Salud Carlos III, 28029 Madrid, Spain; 4 Department of Microbiology, Hospital Universitario Son Espases, Health Research Institute of the Balearic Islands (IdISBa), 07120 Palma de Mallorca, Spain; 5 Department of Clinical Microbiology, Biomedical Diagnostic Center, Hospital Clinic, 08036 Barcelona, Spain

## 1. Error in Figure

In the original publication [1], there was a mistake in Figure 1 as published. The molecule represented in Figure 1 is inaccurate, as the order of the amino acids is reversed, and the amine group of D-Pro(NH_2_) must be eliminated. The corrected Figure 1 appears below. 

## 2. Text Correction

There was an error in the original publication [1]. The sequence of MV6 specified in the Section “2.1. MV6 Structure” is incorrect. It includes an additional amino group in the first D-Proline composing the peptide. Additionally, the correct pattern of MV6 is Arg-D-Pro-Trp, not Trp-D-Pro-Arg.

A correction has been made to Section 2. Results, “2.1. MV6 Structure”, first paragraph:

The MV6 cyclic peptide was selected for further study against *A. baumannii* from a synthetic library of 28 cyclic peptides following a small-scale “shot in the dark” approach in which each peptide was tested in combination with various antibiotics and bacterial species. Its structure consists of six amino acids, two arginine residues (Arg), two D-proline residues (D-Pro), and two tryptophan residues (Trp), arranged in a cyclic configuration. The final structure is &Arg-D-Pro-Trp-Arg-D-Pro-Trp& (Figure 1).

The authors state that the scientific conclusions are unaffected. This correction was approved by the Academic Editor. The original publication has also been updated.

## Figures and Tables

**Figure 1:**
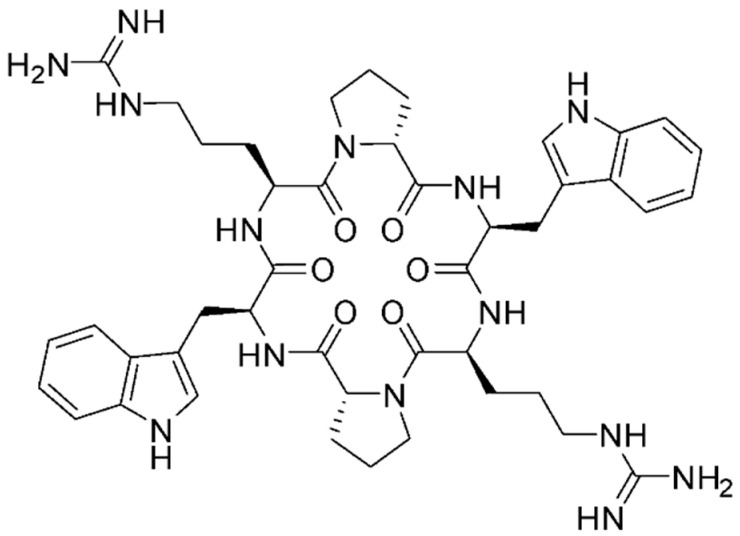
Chemical structure of MV6 cyclic peptide.
